# Effects of dietary piperine on growth, hemolymph chemistry, body composition, antioxidant state, immune response, and resistance against *Vibrio parahemolyticus* in whiteleg shrimp (*Litopenaeus vannamei*)

**DOI:** 10.5455/javar.2024.k850

**Published:** 2024-12-28

**Authors:** Najah M. Albaqami

**Affiliations:** Department of Biological Sciences, Faculty of Sciences, King Abdulaziz University, Jeddah, Saudi Arabia

**Keywords:** Whiteleg shrimp, dietary piperine, health, *V. parahemolyticus*, immunity

## Abstract

**Objective:**

This study investigated the effects of dietary piperine (PIP) supplementation on feed efficiency, growth performance, digestive enzyme activity, hemolymph biochemistry, antioxidant and immune responses, and disease resistance in whiteleg shrimp (*Litopenaeus vannamei*) challenged with *Vibrio parahemolyticus*.

**Materials and Methods:**

A total of 320 shrimps (4.38 gm ± 0.2 gm) were randomly distributed into four treatments and fed a basal diet or supplemented with 0, 0.5 (PIP0.5), 1 (PIP1.0), and 2 (PIP2) gm/kg of diet.

**Results:**

The dietary inclusion of PIP significantly improved growth performance, blood proteins, and efficiency, resulting in the best results in the PIP2 group. Diets containing PIP led to significant improvements in crude protein and lipid content while also significantly reducing moisture content in a quadratic-dependent manner (*p* < 0.05).

Shrimp in the PIP1 and PIP2 groups exhibited higher total protein and albumin levels compared to the free-PIP and PIP0.5 groups (*p* < 0.05). Shrimp-fed PIP-enriched diets showed lower lipid profiles (total cholesterol, triglycerides, and low-density lipoprotein) and liver enzymes (gamma-glutamyl transferase and lactate dehydrogenase) in a quadratic trend (*p* < 0.05) compared to the control diet. Shrimp-fed diets containing PIPs exhibited a significant quadratic increase in digestive enzyme activity compared to those without PIPs (*p* < 0.05). The inclusion of PIP in the diet significantly enhanced antioxidant enzymes and reduced malondialdehyde levels, as well as the inflammatory response (Interleukin 4, Interferon-gamma, and LYZ) in shrimp.

**Conclusion:**

These findings suggest that dietary PIP supplementation holds promise as a feed additive for enhancing growth, immunity, and disease resistance in whiteleg shrimp.

## Introduction

Aquaculture is the fastest growing sector in animal-origin food production, contributing more than 50% of the fish consumed globally [1]. The extensive aquaculture industry is growing rapidly, but there are several limitations to expanding this sector, such as disease infections and the cost of feed. Shrimp farming is a significant sector in the aquaculture industry, playing a vital role in the global economy. In 2022, approximately 4.3 million tons of shrimp were produced globally, of which 70% of the total production was recorded in the global market [2]. Whiteleg shrimp (*L. vannamei*) remains the world’s most broadly cultivated shrimp species. However, in recent years, there has been a slowdown in shrimp growth due to outbreaks of infectious diseases such as acute hepatopancreatic necrosis disease (AHPND) caused by *Vibrio parahaemolyticus* [3].

*Vibrio parahaemolyticus* is a significant pathogen in shrimp aquaculture, causing substantial economic losses worldwide. It primarily affects the hepatopancreas (liver and pancreas) of shrimp, leading to a condition known as AHPND [3]. The dietary inclusion of certain immunostimulatory phytochemicals can enhance resistance to *V. parahaemolyticus* in shrimp [2]. However, this disease is still one of the major causes of mortality in the shrimp industry. To address these challenges, researchers have developed various nutritional strategies to protect shrimp farms and enhance their disease resistance [4,5]. One popular approach involves the use of immunostimulant feed additives. Many plant-derived compounds and extracts, known as phytochemicals, have shown promise as potent immunostimulants for shrimp [4,6]. These phytochemicals have been effective in enhancing shrimp resistance to pathogens like *V. parahaemolyticus* due to their rich content of active compounds such as alkaloids, phenolics, tannins, saponins, glycosides, and terpenoids, with promising antioxidant, anti-inflammatory, and antimicrobial properties [4,7]. Beyond their immune-boosting properties, phytochemicals have also been shown to improve shrimp growth performance and feed efficiency [7]. Plant extracts are rich in alkaloid molecules with various therapeutic uses, including antioxidant, hepatoprotective, and anti-tumor effects [8,9].

A key characteristic of alkaloids is their ability to function as either hydrogen bond acceptors or donors, depending on the specific amine functionality present within their structure. This property is crucial for the interactions between biological targets (enzymes, proteins, and receptors) and drug molecules, making them more safe, effective, and applicable in various aquatic animal aspects [10]. Compared to synthetic molecules, plant-derived constituents are more biodegradable, environmentally friendly, and cost-effective. They have also been shown to promote growth, appetite, digestion, immunity, and disease resistance in aquatic animals [4,7,11,12].

Piperine (PIP), an alkaloid derived from various *Piperaceae* plants, possesses a unique chemical structure featuring an aromatic ring with a methylenedioxy bond [9], a conjugated dienone scheme, and a piperidine ring linked by an amide bond. This structural configuration is believed to contribute to PIP’s diverse bioactivities [8]. PIP can regulate glucose and lipid metabolism, mitigate oxidative stress, and enhance immune function in mammals through various biochemical and physiological methods [6,8,11]. Moreover, research in livestock and poultry has shown that PIP supplementation can improve growth indices, feed utilization, immune function, and gut health [13,14,15]. Specifically, black pepper extracts have exhibited inhibitory effects against various bacteria. Dietary PIP addition in *Labeo rohita* and *Epinephelus tauvina* has been shown to improve survival rates when challenged with *Aeromonas hydrophila* and *Vibrio harveyi* infections [11]. However, the application of PIP in marine fish such as shrimp is relatively unexplored, limiting its potential as a novel feed additive in aquaculture.

Due to the various biological activities of PIP, we hypothesized that the dietary administration of PIP would improve the growth, health hemolymph, immune ability, antioxidant function, and disease resistance against *V. parahaemolyticus* in shrimp. Therefore, this study aimed to assess the impact of dietary PIP addition on growth, feed competence, hemolymph chemistry, immunity, antioxidant capacity, and disease resistance against *V. parahaemolyticus* in shrimp.

## Materials and Methods

### Ethical statement

This experiment was approved by the Department of Biological Sciences, Zoology, King Abdulaziz University, Saudi Arabia by the U.K. Animals (Scientific Procedures) Act, 1986 and associated guidelines, EU Directive 2010/63/EU for animal experiments, the National Research Council’s Guide for the Care and Use of Laboratory Animals (NIH Publications No. 8023, revised 1978) and in compliance with the ARRIVE guidelines.

### Experimental design and setup

A total of 320 healthy juvenile *L. vannamei* weighing 4.38 ± 0.2 gm were used to investigate the potential effects of dietary PIP on growth, immunity, hemolymph biochemistry, and antioxidant status in shrimp over 90 days. Before the experiment, the shrimp was acclimated in hapas for 1 week. The hapa size used was 1 m^3^ (width 1 × length 1 × depth 1 m). The mesh size ranges from 8 mm (5/16) depending on the initial size of fry/fingerling stocked. Sixteen hapas were utilized in the experiment with a stocking density of 20 shrimps per hapa. Shrimps were provided by the Sustainable Agriculture Production Research Group, Department of Biological Sciences, Zoology, King Abdulaziz University, Saudi Arabia. The shrimp were randomly distributed among the 16 hapas, divided into four experimental groups. Each group consisted of four hapas with 80 shrimps in each group, totaling four replicates. The experimental groups were as follows: the control group (PIP0), where shrimps were fed a basal diet without any supplement. The second, third, and fourth groups were fed a basal diet supplemented with 0.5 (PIP0.5), 1 (PIP1), or 2 (PIP2) gm of PIP per kg of diet, respectively. The PIP dose was determined based on previous studies [11,12]. The diet formula and proximate composition are stated in Table 1. Shrimps were hand-fed three times daily at 8.30, 13:00, and 16:30 h for three months at 4% (first 6 weeks) or 5% (last weeks) of their average body weight. The feed was provided by ARASCO company in Riyadh, Saudi Arabia. The water physiochemical features (mean ± SD) were reserved at the optimal conditions for shrimp; hapa dimensions: 1 × 1 × 1 m, pH (7.63 ± 0.04), water temperature (24.12°C ± 0.21°C), salinity (26.7 ± 0.1), 6.24–7.20 mgl^−1^ for dissolved oxygen values, 0.02–0.04 mgl^−1^ for nitrite, 7.60–8.2 for pH values, 0.02–0.03 mgl^−1^ for nitrates, and 0.3–0.5 mgl^−1^ for NH_4_. The proximate assessments of the diets used are explained in Table 1, and the chemical components of the prepared diet models were assessed according to the procedures outlined in [16].

**Table 1.** The dietary composition of shrimp (gm/kg) used in this experiment.

Components	Experimental diets			
	PIP0	PIP0.5	PIP1	PIP2
Fish meal	300.0	300.0	300.0	300.0
Shrimp meal	250.0	250.0	250.0	250.0
Rice bran	70.0	70.0	70.0	70.0
Wheat flour	120.0	118	117.5	117.0
Soybean meal	150.0	150.0	150.0	150.0
CMC	10.0	10.0	10.0	10.0
Fish oil	60.0	60.0	60.0	60.0
Piperine	0.0	0.5	1.0	2.0
Vitamins mixture	20.0	20.0	20.0	20.0
Mineral mixture	20.0	20.0	20.0	20.0
total	1,000.0	1,000.0	1,000.0	1,000.0
Proximate composition of diets (%)				
Dry matter	90.64	90.83	91.31	90.60
Crude fiber	1.74	1.57	1.32	1.60
Crude fat	10.96	10.80	10.85	10.87
Crude protein (N × 6.25)	38.80	38.76	38.76	38.80
Moisture	9.35	9.02	8.76	9.30
Ash	6.15	6.62	7.11	6.42
Carbohydrate (NFE)	32.975	33.087	32.291	33.017
Gross energy kcal/100 gm	459.467	458.56	459.22	457.61

*NFE: nitrogen-free extract; CMC: carboxymethylcellulose; PIP0, PIP0.5, PIP1, and PIP2 are a basal diet and different amounts of diet PIP (0, 0.5, 1, and 2 gm per kilogram).

### Growth indices

The growth performance of each group was assessed based on the methods of [4]. The body weights were measured using an electronic digital balance (±0.01 gm). The shrimps were weighed at the initiation and the end of the experiment and considered as initial body weight (IBW, gm/shrimp) and final body weight (FBW, g/shrimp), respectively. Body weight gain (BWG) = FBW (gm) − IBW (gm). Average weight gain (AWG, gm/shrimp/day) = [(FBW − IBW) − IBW]/duration period. Total feed intake (TFI, gm/shrimp): the amount of the supplemented diet throughout the study period per shrimp. Feed conversion ratio (FCR) = TFI (gm)/weight gain (gm). Survival rate (SR) = (Z/X) × 100, where Z is the quantity of remaining shrimp and X is the sum of the initial shrimp stock. Specific growth rate (SGR) = 100 × (lnW2 − lnW1)/T, where ln is the natural logarithm, W2 is the final weight (gm), W1 is the initial weight, and T is the number of days of the feeding period.

### Hemolymph biochemistry

Twelve whiteleg shrimps (*L. vannamei*) from each treatment were utilized for hemolymph gathering. Approximately 250 μl of a hemolymph sample was collected from the base of the third walking leg of each shrimp using a sterile syringe (1 ml) comprising 750 μl of precooled (4°C) anticoagulant solution (450-mM NaCl, 10-mM KCl, 0.114-M trisodium citrate, and 10-mM HEPES at pH 7.4) [17]. The mixture of hemolymph and anticoagulant was employed to determine biochemical parameters, immunity, antioxidant levels, and inflammatory response. Total protein, albumin, globulin, gamma-glutamyl transferase (GGT), lactate dehydrogenase (LDH), total cholesterol (TC), triglycerides (TGs), low-density lipoprotein (LDL), and high-density lipoprotein (HDL) were assessed using commercial kits that were acquired from Biodiagnostic Biotechnology Co. (Giza, Egypt).

### Digestive enzymes

The levels of digestive enzymes (protease, lipase, and amylase) in the hemolymph were assessed using the method illustrated in [18]. The protease (MBS706715), amylase (MBS077098), and lipase (MBS019742) enzymes were measured with commercial kits through a spectrophotometric colorimetric approach at 405 nm, 450 nm, and 570 nm, respectively, following the protocols outlined by Sontakke et al. [19] and [20]. The commercial kits were supplied by MyBioSource company (San Diego, USA).

### Whole-body composition assessments

The proximate composition of the whole body was analyzed using typical approaches as described in the study by AOAC [21]. The whole-body composition of the shrimp was established using conventional techniques following the method outlined in [22]. Protein content was considered by applying the Kjeldahl procedure [5], while the crude lipid was calculated through ether extraction using the Soxtec approach [23]. Ash content was established by incinerating the sample in a muffle oven at 550°C for 4 h. Moisture content was determined by drying the sample in a hot air oven at 105°C until a constant weight was achieved. Crude fiber was analyzed using acid digestion with the Fibra Plus apparatus.

### Antioxidant activities and pro-inflammatory cytokines

Antioxidant capacity was evaluated by measuring glutathione peroxidase (GPx; K762-100), total antioxidant capacity (TAC; K274-100), and superoxide dismutase (SOD; Catalog #K335-100) activity using commercially available colorimetric assay kits (Biovision, Inc., California, USA). Malondialdehyde (MDA) levels, an indicator of lipid peroxidation, were also determined using a colorimetric assay kit from the same supplier. All assays were performed according to the manufacturer’s instructions.

Interleukin 4 (IL-4, MBS281557) and Interferon-gamma (IFN‐γ, MBS702530) were assessed using commercial kits provided by MyBioSource (Santigho, USA). Lysozyme activity was measured using a turbidimetric assay based on the lytic function of lysozyme on *Micrococcus lysodiekticus *bacteria [24]. Hemolymph (20 μl) was mixed with a bacterial suspension (0.75 mg/ml in 0.1-M sodium phosphate buffer, pH 6.4) in a 200-μl mixture and incubated at 37°C. The decrease in optical density was monitored at 570 nm. A standard curve was prepared using hen egg white lysozyme from Sigma-Aldrich. Results are expressed as μg/ml.

### Challenge trial

After the feeding trial ended, a total of 60 shrimps were randomly selected for the challenge test (20 from each group) with *V. parahaemolyticus* and placed in 12 transparent plastic aquariums (55 × 35 × 30 cm^3^). All shrimps were exposed to a bacterial suspension containing virulent *V. parahaemolyticus* at a concentration of 1 × 10^5^ CFU ml^−1^ (LC_50_) [25] at 28°C through immersion in seawater mixed with the bacterial suspension without renewing the water for 48 h. Subsequently, the aquarium tanks were replaced with 50% fresh seawater, and the shrimps were fed with experimental diets twice daily for 15 days. Daily shrimp mortality was monitored, and the cumulative mortality percentages (MR) were calculated every three days (0, 3, 6, 9, 12, and 15) using the formula MR% = number of dead shrimps at the different times of the experiment/number of shrimps at the beginning of the experiment *100.

### Statistical analysis

To ensure data normality and homogeneity of variance, Shapiro–Wilk and Levene’s tests were conducted. Statistical analyses were performed using SPSS 25.0 (SPSS Inc., Chicago, IL, USA) with a mixed-effects model (PROC MIXED). Orthogonal contrast statements were used to assess linear and quadratic dose–response relationships for each dependent variable across different PIP levels (0-, 0.5-, 1-, and 2-gm/kg diet). Significant relationships were further analyzed using regression analysis to determine the optimal PIP dosage. Statistical significance was set at a *p*-value of less than 0.05. Duncan’s multiple range test was applied for post hoc comparisons.

## Results

### Effects on growth indices

Dietary PIP supplementation significantly improved the FBW, BWG, AWG, SR, and SGR while decreasing the FCR and TFI in a quadratic-dependent manner (Table 2). The best consequences for FBW were detected in the PIP2 group. The PIP0.5 and PIP1 groups exhibited higher BWG compared to the free-PIP diet (*p* < 0.05). TFI and FCR were significantly improved by increasing the levels of PIP in shrimp diets (*p* < 0.05). A quadratic relationship showed that shrimp-fed diets with PIP had higher SR, SGR, and FBW than those fed a free-PIP diet (*p* < 0.05).

### Effects on whole-body composition

Shrimp-fed diets with PIP (0.5–2 gm/kg diet) significantly improved the crude protein and lipid content while significantly decreasing the moisture content in a quadratic-dependent manner (Table 3). There was no significant consequence of dietary PIP on the ash content (*p* > 0.05).

**Table 2.** Impacts of dietary PIP (0.5-, 1-, and 2-gm/kg diet) on the growth performance and feed utilization of Pacific shrimp.

Item^1^	Treatments				*p*-value		
	PIP0	PIP0.5	PIP1	PIP2	TRTs	LIN	QAU
IBW, gm	4.40 ± 0.09	4.41 ± 0.05	4.38 ± 0.13	4.35 ± 0.07	0.77	0.38	0.65
FBW, gm	25.69 ± 0.61^b^	28.17 ± 0.14^a^	28.22 ± 0.39^a^	29.44 ± 0.28^a^	0.001	0.001	0.02
BWG, gm	21.29 ± 0.70^c^	23.75 ± 0.12^b^	23.84 ± 0.49^b^	25.09 ± 0.22^a^	0.001	0.001	0.05
AWG, gm	0.23 ± 0.01^c^	0.26 ± 0.01^b^	0.26 ± 0.01^b^	0.28 ± 0.01a	0.001	0.001	0.05
TFI, gm	38.33 ± 1.53^a^	36.00 ± 1.37^b^	35.00 ± 1.20^b^	35.33 ± 1.15^b^	0.03	0.01	0.09
FCR, gm feed/gm gain	1.80 ± 0.10^a^	1.52 ± 0.05^b^	1.47 ± 0.05^b^	1.41 ± 0.05^c^	0.001	0.001	0.018
SGR, %	1.67 ± 0.05^a^	1.90 ± 0.03^a^	2.00 ± 0.02^a^	1.99 ± 0.03^a^	0.001	0.001	0.001
SR, %	75.00 ± 1.00^b^	81.77 ± 0.67^a^	82.64 ± 0.85^a^	82.78 ± 0.60^a^	0.001	0.001	0.001

Final body weight (FBW), initial body weight (IBW), body weight gain (BWG), average weight gain (AWG), total feed intake (TFI), feed conversion ratio (FCR), superficial growth rate (SGR), and survival rate (SR). Shrimp-fed diet supplemented with 0 (PIP0), 0.5 (PIP0.5), 1 (PIP1), and 2 (PIP) gm/kg diet. Lin, linear regression; TRTs, treatment; quad, quadratic regression. ^a,b,c ^Means in the same column showing various superscripts are significantly different at *p* < 0.05.

**Table 3.** Impacts of dietary PIP (0.5-, 1-, and 2-gm/kg diet) on the carcass traits in Pacific white shrimp (*L. vannamei*).

Item	Treatments				*p*-value		
	PIP0	PIP0.5	PIP1	PIP2	TRTs	LIN	QAU
Crude protein, %	15.97 ± 0.21^b^	16.18 ± 0.06^a^	16.13 ± 0.05^a^	16.10 ± 0.03^a^	0.03	0.026	0.019
Crude lipid, %	1.85 ± 0.04^b^	2.00 ± 0.02^a^	2.05 ± 0.06^a^	2.03 ± 0.07^a^	0.004	0.002	0.019
Ash, %	3.06 ± 0.04	3.08 ± 0.04	3.10 ± 0.11	3.07 ± 0.03	0.947	0.935	1.000
Moisture, %	79.12 ± 0.21^a^	78.73 ± 0.25^b^	78.68 ± 0.27^b^	78.80 ± 0.04^b^	0.099	0.104	0.049

Shrimp-fed diet supplemented with 0 (PIP0), 0.5 (PIP0.5), 1 (PIP1), and 2 (PIP2) gm/kg diet. ^a,b^Means in the same column exhibiting diverse superscripts are significantly different at *p* < 0.05. Lin, linear regression; TRTs, treatment; quad, quadratic regression.

### Effects on hemolymph chemistry

Shrimp in groups PIP1 and PIP2 had higher total protein and albumin levels than the free-PIP and PIP0.5 groups (*p* < 0.05, Table 4). Globulin was not affected by the diet (*p* > 0.05). PIP0.5 group exhibited intermediate values with significant differences among other groups (*p* < 0.05). Feeding shrimp PIP in their diets tended to reduce the lipid profile (TC, TG, and LDH) and liver enzymes (GGT and LDH) in the hemolymph in a quadratic trend (*p* < 0.05). HDL levels were quadratically improved due to the PIP dietary administration (*p* < 0.05). Dietary PIP improved blood protein levels, regulated lipid profiles, and enhanced liver function in shrimp.

### Effects on digestive enzymes

Feeding shrimp diets enriched with PIP resulted in a significant quadratic improvement in digestive enzymes compared to the free-PIP diets (*p* < 0.05, Table 5). The best results for amylase, lipase, and protease were observed in the PIP1 group in a quadratic dose-dependent manner (*p* < 0.05). There was no noteworthy variation between the PIP0.5 and PIP2 groups for amylase, but they had a significant effect compared to the control diet. It is noted that higher PIP levels indicated significantly worse levels of lipase than other groups (*p* < 0.05). Feeding shrimp with 0.5 or 2 gm of PIP/kg diet significantly improved protease activity (*p* < 0.05).

### Effects on antioxidant/inflammatory responses

Dietary PIP inclusion significantly enhanced the antioxidant enzymes and reduced MDA levels, as well as the inflammatory response (IL-4, IFN‐γ, and LYZ) in shrimp (Table 6). Shrimp given 1 or 2 gm of PIP/kg diet showed a significant increase in TAC and GPx in a linear trend (*p* < 0.05). In contrast, all PIP-supplemented diets resulted in a significant reduction in MDA levels and lysozyme activity (*p* < 0.05) in a quadratic manner. A quadratic regression analysis indicated that shrimp in the PIP1 group had the highest SOD levels and the lowest IFN‐γ levels compared to other groups (*p* < 0.05). SOD was similar between the control and PIP0.5 groups (*p* > 0.05). The PIP2 group displayed intermediate values for IL-4 and IFN‐γ compared to other groups. Adding PIP to shrimp diets improved antioxidant status and reduced stress-related markers in a dose-dependent way (*p* < 0.05).

**Table 4.** Impacts of dietary PIP (0.5-, 1-, and 2-gm/kg diet) on the hemolymph chemistry in Pacific white shrimp (*L. vannamei*).

Item	Treatments				*p*-value		
	PIP0	PIP0.5	PIP1	PIP2	TRTs	LIN	QAU
TP, gm/dl	2.82 ± 0.16^c^	3.33 ± 0.28^b^	4.54 ± 0.29^a^	4.24 ± 0.12^a^	0.001	0.001	0.014
ALB, gm/dl	1.68 ± 0.17^c^	2.18 ± 0.27^b^	3.32 ± 0.35^a^	3.08 ± 0.10^a^	0.001	0.001	0.028
GLO, gm/dl	1.14 ± 0.02	1.15 ± 0.10	1.22 ± 0.11	1.16 ± 0.03	0.642	0.546	0.488
GGT, IU/l	62.52 ± 2.10^a^	49.04 ± 2.30^b^	36.07 ± 1.17^b^	39.16 ± 1.01^b^	0.001	0.001	0.001
LDH, IU/l	122.13 ± 3.44^a^	91.07 ± 3.15^b^	62.73 ± 2.35^b^	61.77 ± 3.23^b^	0.001	0.001	0.001
TC, mg/dl	92.37 ± 1.65^a^	62.74 ± 1.10^b^	62.53 ± 1.16^b^	60.76 ± 1.56^b^	0.001	0.001	0.001
TG, mg/dl	105.40 ± 4.57^a^	72.25 ± 1.21^b^	72.40 ± 1.38^b^	72.20 ± 1.49^b^	0.001	0.001	0.001
LDL, mg/dl	42.98 ± 1.91^a^	36.30 ± 0.53^b^	34.57 ± 0.14^b^	35.35 ± 1.19^b^	0.001	0.004	0.514
HDL, mg/dl	21.74 ± 0.95^b^	34.03 ± 1.87^a^	32.85 ± 1.68^a^	33.25 ± 2.14^a^	0.001	0.001	0.001

Total protein (TP), globulin (GLO), albumin (ALB), gamma-glutamyl transferase (GGT), lactate dehydrogenase (LDH), total cholesterol (TC), triglycerides (TG), low-density lipoprotein (LDL), and high-density lipoprotein (HDL). Shrimp-fed diet supplemented with 0 (PIP0), 0.5 (PIP0.5), 1 (PIP1), and 2 (PIP2) gm/kg diet. ^a,b,c ^Means in the same column showing diverse superscripts are significantly different at *p* < 0.05. Lin, linear regression; TRTs, treatment; quad, quadratic regression.

**Table 5.** Impacts of dietary PIP (0.5-, 1-, and 2-gm/kg diet) on the digestive enzymes in Pacific white shrimp (*L. vannamei*).

Item	Treatments				*p* value		
	PIP0	PIP0.5	PIP1	PIP2	TRTs	LIN	QAU
Amylase, IU	179.96 ± 2.13^c^	203.89 ± 3.23^b^	222.63 ± 2.26^a^	209.51 ± 2.35^b^	0.001	0.001	0.001
Lipase, IU	227.15 ± 3.58^c^	237.58 ± 3.78^b^	244.00 ± 3.61^a^	220.83 ± 3.22^d^	0.001	0.192	0.001
Protease, IU	166.50 ± 5.38^d^	192.89 ± 2.53^c^	217.34 ± 2.53^a^	210.10 ± 3.39^b^	0.001	0.001	0.001

Shrimp-fed diet supplemented with 0 (PIP0), 0.5 (PIP0.5), 1 (PIP1), and 2 (PIP2) gm/kg diet. ^a,b,c,d ^Means in the same column showing diverse superscripts are significantly different at *p* < 0.05. Lin, linear regression; TRTs, treatment; quad, quadratic regression.

**Table 6.** Impacts of dietary PIP (0.5-, 1-, and 2-gm/kg diet) on the redox state and pro-inflammatory cytokines in Pacific white shrimp (*L. vannamei*).

Item	Treatments				*p*-value		
	PIP0	PIP0.5	PIP1	PIP2	TRTs	LIN	QAU
Antioxidant-related markers							
TAC, nmol/ml	3.81 ± 0.38^c^	4.12 ± 0.24^b^	5.22 ± 0.30^a^	5.03 ± 0.14^a^	0.001	0.001	0.159
GPx, nmol/ml	2.68 ± 0.01^c^	2.77 ± 0.01^b^	3.27 ± 0.27^a^	3.35 ± 0.18^a^	0.001	0.001	0.940
SOD, nmol/ml	1.23 ± 0.10^c^	1.30 ± 0.11^c^	1.73 ± 0.11^a^	1.50 ± 0.12^b^	0.002	0.002	0.043
Stress-related markers							
MDA, nmol/ml	4.18 ± 0.81^a^	1.25 ± 0.03^b^	1.25 ± 0.01^b^	1.25 ± 0.01^b^	0.001	0.001	0.001
IL-4, ng/ml	5.27 ± 0.09^a^	3.59 ± 0.03^c^	3.50 ± 0.04^c^	4.63 ± 0.21^b^	0.001	0.001	0.001
IFN‐γ, ng/ml	3.10 ± 0.09^a^	1.83 ± 0.16^b^	1.52 ± 0.10^c^	1.79 ± 0.06^b^	0.001	0.001	0.001
LYZ, μU/ml	4.51 ± 0.38^a^	3.19 ± 0.11^b^	3.24 ± 0.05^b^	3.25 ± 0.10^b^	0.001	0.001	0.001

Total antioxidant capacity (TAC), glutathione peroxidase (GPx), superoxide dismutase (SOD), malondialdehyde (MDA), interleukin -4 (IL-4), interferon-gamma (IFN‐γ), and lysozyme activity (LYZ). Shrimp-fed diet supplemented with 0 (PIP0), 0.5 (PIP0.5), 1 (PIP1), and 2 (PIP2) gm/kg diet. ^a,b,c ^Means in the same column showing diverse superscripts are significantly different at *p *< 0.05. Lin, linear regression; TRTs, treatment; quad, quadratic regression

### Challenge experiment

**Figure 1.**
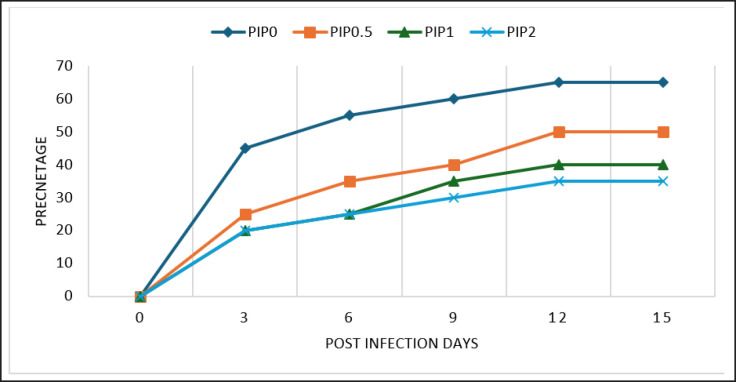
The morality of shrimp after infection with *V. parahemolyticus* for 15 days and fed diets with 0 (PIP0), 0.5 (PIP0.5), 1 (PIP1), and 2 (PIP2) gm of PIP/kg diet.

We used 20 shrimps in each group for this challenge test. After 3 days post-infection, the mortality rate of the control group was 45% (9/20), while at the end of the experiment, it was 65% (13/20). Shrimps fed PIP at 0.5-, 1-, or 2-gm/kg diet and infected with *V. parahemolyticus* had mortality rates of 25% (5/20), 20% (4/20), and 20% (4/20), respectively. The highest mortality rate was observed in the control group, while the lowest mortality rate was shown in shrimp fed a diet supplemented with 2 gm of PIP/kg (Fig. 1).

## Discussion

**Figure 2.**
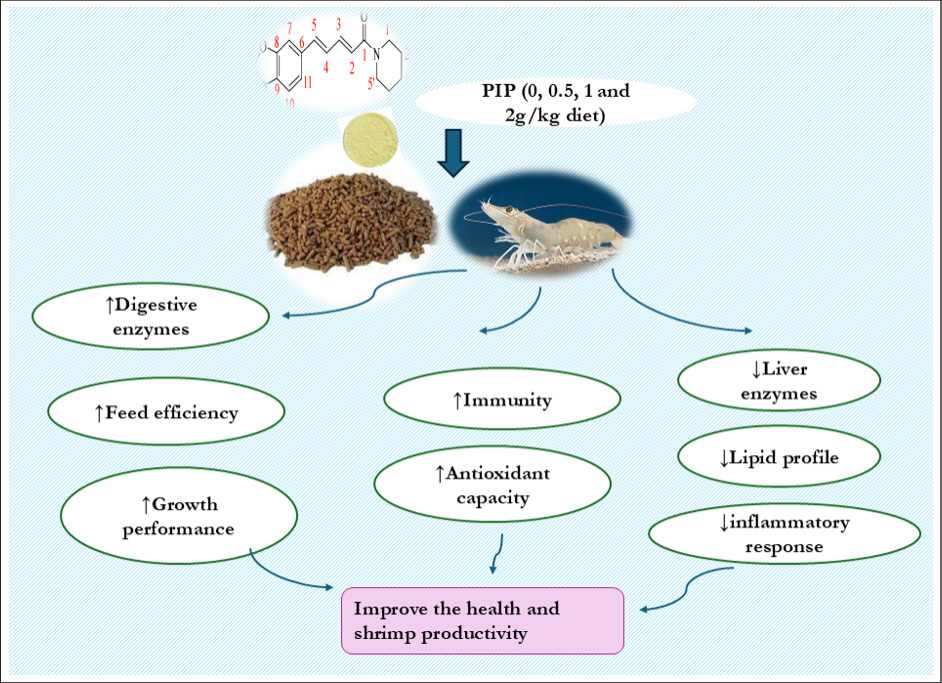
The mode of action of dietary PIP on shrimp physiology involves stimulating digestive enzymes, promoting feed efficiency, and enhancing growth performance. PIP can also promote antioxidant and immune functions, reduce lipid profiles and liver enzymes, and decrease inflammatory responses during the growth phase.

Phytochemicals have been widely used in aquaculture to enhance growth, blood health, and overall physiological status and improve disease resistance. PIP, a phytochemical alkaloid extracted from black pepper seeds (*Piper nigrum*), has shown a wide range of biological activities, comprising antioxidants, anti-tumor, and anti-inflammatory effects [8,11], as well as potential benefits in combating obesity [12]. In this study, the dietary inclusion of PIP led to significant improvements in growth features, feed efficiency, immunity, and disease resistance against *V. parahemolyticus*. These results were supported by increased secretion of digestive enzymes, improved antioxidant status (SOD, GPx, and TAC), reduced oxidative stress MDA, and inflammatory responses (IL-4, INFG, and lysosome activity) in shrimp (Fig. 2).

Numerous searches have established a strong correlation between the structural properties of PIP and its derivatives and their diverse biological actions, with antioxidant and anti-inflammatory consequences [8,9]. Prior inquiries have employed various methods to assess the antioxidant activity of numerous solvent extracts, piperic acid, and PIP, with synthetic piperic acid demonstrating the highest potency [26]. Furthermore, a novel starch piperinic ester with anti-hyperlipidemic properties was produced by linking the carboxyl group of piperic acid to the hydroxyl group of starch [27]. Based on these findings, it is plausible that the observed beneficial effects of PIP in shrimp diets, such as improved growth, enhanced health, and immune function, may be attributed to the antioxidant, immunomodulatory effect, and antimicrobial properties of PIP.

Robust growth performance is essential for maximizing economic returns in shrimp aquaculture. In this study, dietary inclusion of PIP (0.5–2 gm/kg) significantly improved the FBW, SGR, and FCR (decreased the values). However, the addition of PIP significantly decreased the TFI, indicating an improvement in FCR in shrimps. These results are consistent with previous findings in many aquatic fish, including *Cyprinus carpio* [28], large yellow croaker (*Larimichthys crocea*) [12], olive flounder (*Paralichthys olivaceus*) [29], red seabream (*P. major*) [30], and *L. rohita* [11].

According to previous studies, PIP can quadratically increase the growth indices and decrease the FCR ratio of shrimp, thereby improving their growth performance. These improvements were accomplished by increasing the secretion of digestive enzymes. Previous research has shown that PIP can enhance the growth performance of fish by stimulating nutrient digestion and absorption [8,9]. Similarly, studies on common carp (*C. carpio*), olive flounder *(P. olivaceus*), and Pacific white shrimp (*L. vannamei*) have demonstrated that PIP can improve growth indices by enhancing nutrient absorption in aquaculture species [11,12,28–30].

The observed enhancement in growth performance is attributed to improved nutrient digestibility and absorption. This study demonstrated that PIP (0.5- and 1-gm/kg diet) can accelerate the secretion of digestive enzymes, including lipase, amylase, and protease, thereby improving nutrient absorption and utilization in shrimp. The study revealed significantly higher lipase activity in large yellow croaker [31,32] fed a diet containing 25-mg/kg PIP compared to the control and other groups. This finding aligns with previous research on large yellow croaker, which suggests that PIP may enhance lipid metabolism, leading to increased lipase activity [31]. These findings corroborate previous research by Abdelnour et al. [33], who demonstrated that adding 1 or 2 ml of red pepper per kg diet significantly enhanced growth and digestive enzyme secretion in rabbits. Similarly, findings in albino rats have revealed that dietary PIP substantially heightened the assembly of digestive enzymes, thereby improving digestive function. Furthermore, according to [12], the activity of digestive enzymes can significantly impact the growth performance of juvenile jundia (*Rhamdia quelen*).

The liver, as the principal metabolic tissue in fish, plays a necessary role in lipid metabolism. Abnormal hepatic lipid deposition is a significant indicator of impaired liver health. Preceding investigations have exhibited that high dietary soybean oil levels can reduce lipid utilization, leading to hepatic lipid accumulation and negatively impacting the health of aquatic animals [11,27,31]. In this case, higher levels of liver enzymes were shown in normal diets, while adding PIP could modulate these elevations and maintain the health of hepatocytes in normal conditions in shrimp. GGT and LDH in shrimp were significantly decreased by dietary PIP inclusion compared to the free-PIP diet.

PIP supplementation in diets can influence lipid deposition by controlling lipid metabolism. In the present study, shrimp supplemented with PIP (1- or 2-gm/kg diet) exhibited substantially lower levels of crude lipid in the liver compared to the control group. Moreover, PIP supplementation significantly decreased hepatic TG and TC levels in large yellow croaker [12]. Recently, similar findings have been observed in large yellow croaker studies [12], where PIP supplementation significantly reduced serum TG, TC, and LDL levels [12,27,30]. Prior animal reports have revealed that PIP can protect against obesity and regulate lipogenic and lipolytic genes, thus lowering body and fat weight in obese mice [27]. Additionally, previous mammalian studies [27] have shown that PIP can significantly reduce serum TG, TC, and LDL levels in mice. Consistent with these results, the current study demonstrated a significant decrease in serum TG and TC levels in a large yellow croaker enhanced with 100 mg/kg of PIP.

Previous research has shown that PIP can inhibit lipid droplet synthesis by downregulating the mRNA expression of peroxisome proliferator-activated receptor gamma (PPARγ) in fat cells [12,34]. According to [12], the addition of 50-mg/kg PIP significantly downregulated the mRNA expression levels of *SREBP-1, FAS,* and *SCD1* in the liver of large yellow croaker, thereby inhibiting lipid synthesis. This finding aligns with previous research on various aquatic fish [12,27,30]. Dietary PIP (0.5/kg diet) increased the intestinal villi length and goblet cell count of olive flounder [29]. Our study shows that adding PIP to the diet of shrimp can boost their immune response and antioxidant capacity as well as reduce the pro-inflammatory response. PIP has been found to reduce lipid peroxidation and increase the activity of GPx in mice, improving their antioxidant capacity [27]. The SOD activity of shrimp was also enhanced by dietary PIP addition. PIP might also be complicated in scavenging excess hydroxyl radicals and superoxide ions, thus protecting shrimp from free radical-mediated damage. Therefore, the immunomodulatory effects of PIP may be accredited to its molecular structure, specifically the piperidine and benzodioxole groups. Additionally, the enhanced lysozyme and antioxidant enzyme activities examined in PIP-treated animals could be due to PIP’s protein-folding ability, as suggested in [11,28]. However, our findings indicate that the antioxidants and immune effects of PIP were linearly improved in a dose-dependent way.

Dietary administration of PIP linearly increased the activities of SOD and CAT in large yellow croaker [12]. Exposure to many environmental issues in shrimp often leads to increased production of reactive oxygen species [31,32]. Antioxidants neutralize free radicals, preventing oxidative stress, lipid peroxidation, and cellular damage.

Liver enzymes, such as GGT and LDH, are indicators of liver health, with elevated levels suggesting liver injury [32,35]. In this study, PIP supplementation at 0.5–2 gm/kg significantly reduced GGT and LDH levels in shrimp, as evidenced in our study and in juvenile large yellow croaker [12], indicating potential liver protection. Previous research in rats has shown that PIP can mitigate oxidative stress by hindering lipid peroxidation and enhancing GPx synthesis and transport, thereby neutralizing excess free radicals and reducing intestinal mucosal oxidative stress. Additionally, PIP can decrease the degradation of antioxidant enzymes and increase their activity, further protecting against oxidative damage. In a mouse liver injury model, PIP supplementation significantly enhanced antioxidant capacity and provided liver protection [36].

In the current study, PIP supplementation at 0.5–2 gm/kg significantly increased TAC and antioxidant enzyme function in the liver of shrimp. Alongside, a significant decrease in MDA levels was observed, suggesting that PIP can enhance antioxidant capacity and reduce lipid peroxidation in shrimp. PIP promoted immune function and significantly reduced inflammation in shrimp, as evidenced by decreased levels of IL-4 and IFN‐γ. Unexpectedly, a significant reduction in lysozyme activity was observed in shrimp-fed diets containing PIP. IL-4 is an important cytokine and has many regulating roles. The addition of PIP significantly reduced the levels of IL-4 and IFN‐γ in shrimp. Acute hepatopancreatic necrosis disease is a severe shrimp disease caused by the V toxins encoded by the Vir plasmid of *V. parahaemolyticus* [8,13,27,35].

Dietary supplementation of PIP at levels up to 1.0 gm/kg can enhance shrimp resistance to *V. parahaemolyticus*. A significant quadratic trend in survival rates suggests an optimal PIP dosage between 0.25 and 1.0 gm/kg for promoting disease resistance. Previous studies have demonstrated the antibacterial properties of various phytochemicals, including azadirachtin, camphor, and curcumin, against different pathogens [11,26,27]. Specifically, black pepper extracts have exhibited inhibitory effects against various bacteria. Dietary PIP addition in *L. rohita* and *E. tauvina* has been shown to improve survival rates when challenged with *A. hydrophila* and *V. harveyi* infections [11]. The immunomodulatory mechanisms of PIP have been investigated in previous *in vivo *and* in vitro* studies. PIP induces plasmolysis in *Escherichia coli* and *Staphylococcus aureus in vitro* [37]. Additionally, PIP inhibits the tricarboxylic acid cycle and delays energy production in these bacteria, leading to cell death. Dietary supplementation with PIP improves antioxidants, immunity, growth performance, feed utilization, and disease resistance in shrimp. This study demonstrates the immunomodulatory effects of PIP in fish, which can provide insights into its potential effects in shrimp.

## Conclusion

Our findings demonstrate that dietary PIP significantly enhances various aspects of shrimp health and growth. In particular, it increased the efficiency of feed, the activity of digestive enzymes, the level of antioxidants, and the immune system’s ability to work while lowering inflammatory and oxidative responses. Notably, PIP proved to be a potent immunostimulant, effectively controlling Vibrio infections. Based on our results, incorporating PIP into shrimp diets at a dosage of 1.0–2.0 gm/kg could offer a promising strategy to boost shrimp production and overall health.

## List of abbreviations

AWG, Average weight gain; FBW, Final body weight; FCR, Feed conversion ratio; GGT, Gamma-glutamyl transferase; GPx, Glutathione peroxidase; HDL, High-density lipoprotein; IBW, Initial body weight; IL-4, Interleukin 4; IFN‐γ, Interferon-gamma; LDH, Lactate dehydrogenase; LDL, Low-density lipoprotein; MDA, Malondialdehyde; PIP, Piperine; SGR, Specific growth rate; SOD, Superoxide dismutase; SPE, Piperinic ester; SR, Survival rate; TAC, Total antioxidant capacity; TC, Total cholesterol; TFI, Total Feed Intake; TG, Triglycerides.
